# Heart rupture repair during huge mediastinal mass resection – case report

**DOI:** 10.1186/s13019-020-01209-9

**Published:** 2020-07-06

**Authors:** Riad Abdel Jail, Mohamad K. Abou Chaar, Obada Al-Qudah, Khalil Abu Zahra, Maysa Al-Hussaini, Azza Gharaibeh

**Affiliations:** 1 Department of Thoracic Oncology, King Hussein Cancer Center, Amman, Jordan; 2 Department of Surgery, King Hussein Cancer Center, Amman, Jordan; 3 Department of Pathology and Laboratory Medicine, King Hussein Cancer Center, Amman, Jordan; 4 Department of Radiology, King Hussein Cancer Center, Amman, Jordan

**Keywords:** Cardiac rupture, Thymoma, Cardiopulmonary resuscitation, Neoadjuvant chemotherapy, Physiotherapy, Case report

## Abstract

**Background:**

Ventricular rupture is rarely described in the literature outside the context of myocardial infarction, infection or neoplasm. It is associated with a high mortality rate due to late presentation and delayed surgical intervention, which involves sutureless or sutured techniques. Comprehensive literature review failed to identify any case of intra-operative right ventricular heart rupture followed by myocardial repair and a complete recovery after a prolonged intensive care unit (ICU) stay.

**Case presentation:**

A 57-year-old previously healthy gentleman presented complaining of a new onset shortness of breath for 2 months. A large mediastinal mass was found on chest imaging and biopsy revealed a thymoma. Patient received a neoadjuvant Cisplatin/Doxorubicin/Cyclophosphamide (CAP) regimen chemotherapy then sternotomy and thymectomy en bloc with anterior pericardium. Post-thymectomy, the patient continued to be hypotensive in recovery despite aggressive fluid resuscitation. He was sent back to theatre, aggressive fluid resuscitation continued, surgical site exploration was done by reopening the sternum, and the bleeding source was identified and controlled, but intraoperative asystole developed. During internal cardiac massage, the right ventricle ruptured with a 3 cm defect which was successfully repaired using a pericardial patch without a bypass machine due to unavailability at our cancer center. The patient remained dependent on mechanical ventilation through tracheostomy for a total of 2 months due to bilateral phrenic nerve paralysis, was discharged from ICU to the surgical floor 66 days after the operation and weaned off ventilator support after 85 days, adequate respiratory and physical rehabilitation followed. Patient is doing very well now with excellent performance, and free of tumor recurrence 30 months after surgery.

**Conclusion:**

Right ventricular rupture is rarely described outside the context of myocardial infarction and valvular heart disease. Tumor proximity to the heart and neoadjuvant cardiotoxic chemotherapy are the proposed causes for precipitating the cardiac rupture in our case. Post-surgical patients who receive early physical rehabilitation and respiratory physiotherapy have improved survival and outcome.

## Introduction

Thymic epithelial tumors originate from typical epithelial cells and include thymomas and thymic carcinomas [1, 2]. Surgery remains the mainstay of treatment in a well-defined anterior mediastinal mass in the thymic bed, with negative tumor markers, absence of lymphadenopathy, and absence of continuity with the thyroid gland [3]. Pericardial invasion is seen in stage IIB [4] which necessitates pericardial resection that might be complicated with intra- or post-operative cardiac ruptures.

## Case presentation

**Fig. 1:**
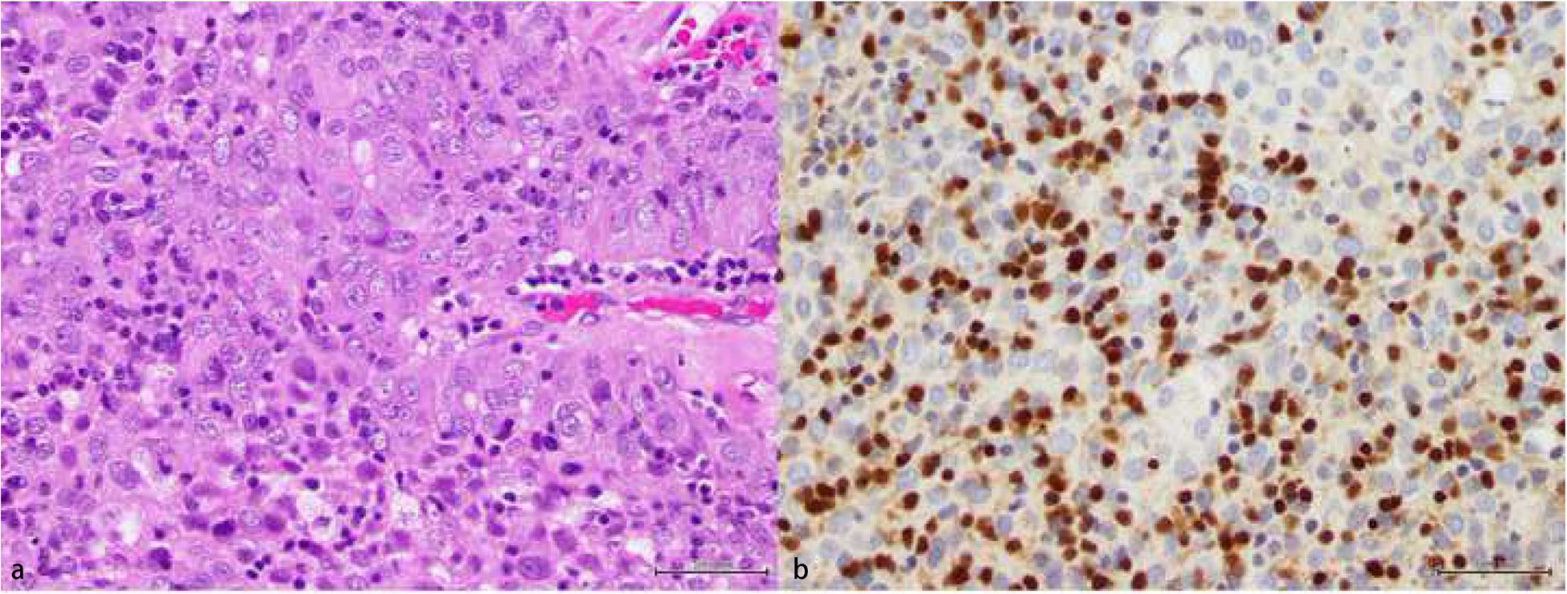
Microscopic images of the resected thymoma. **a**; there is proliferation of squamoid cells with vesicular nuclei and prominent nucleoli. Scattered lymphocytes are seen admixed with the epithelial cells (H&E X40). **b**; the lymphocytes show positive nuclear stain for TdT (X40). Both features are supportive of the diagnosis of thymoma, B3 variant

**Fig. 2:**
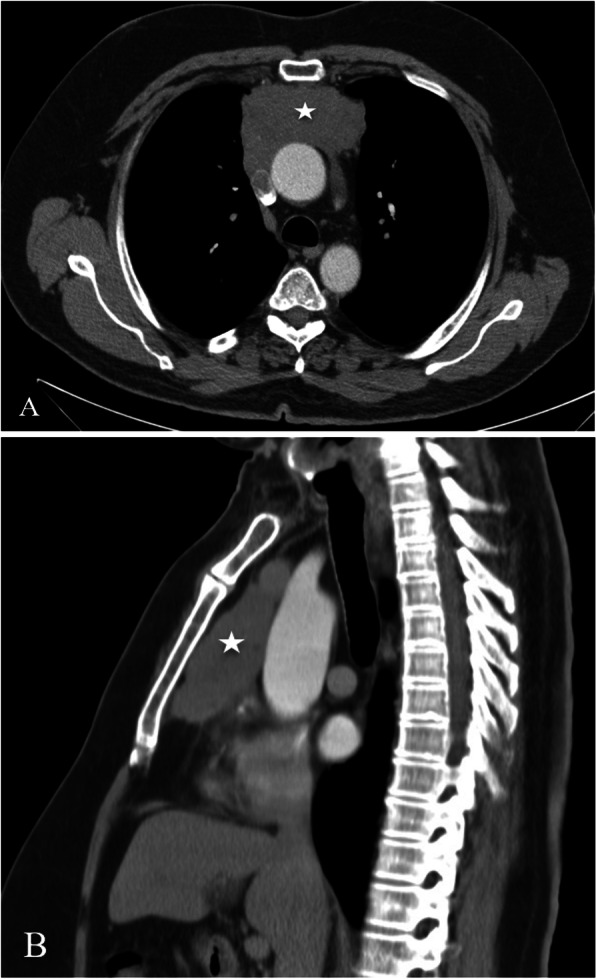
Chest CT scan with contrast, coronal view, mediastinal window showing a lobulated anterior mediastinal soft tissue mass lesion measures 10 X 6 cm (white asterisk) at the level of the aortic arch. The lesion is causing mild compression upon the SVC. However, the SVC is patent. The lesion is inseparable from the anterior wall of the SVC, shows wide area of contact with the ascending aorta, upper pericardium and associated with mild pericardial effusion

A 57-year-old previously healthy gentleman presented to our service complaining of a new onset shortness of breath for 2 months. He initially sought medical attention outside King Hussein Cancer Center (KHCC) where he was found to have a huge mediastinal mass on a chest x-ray from which a biopsy revealed a thymoma (Fig. 1). At KHCC, a chest computed tomography (CT) scan showed a lobulated anterior mediastinal soft tissue mass measuring 11.3 × 7.7 cm at the level of the aortic arch. It appeared to be inseparable from the anterior wall of the superior vena cava (SVC), compressing it mildly but keeping it patent. In addition, it had a wide area of contact with the ascending aorta and upper pericardium. Multiple small nodular soft tissue masses were seen in the anterior epicardial space, mostly representing small lymph nodes. Otherwise no other mediastinal lymphadenopathy was reported (Fig. 2a, b).

A multidisciplinary team explained to the patient and his family the treatment options, which included neoadjuvant chemotherapy and re-assessment of tumor size for potential resectability afterwards. The patient received two cycles of Cisplatin/Doxorubicin/Cyclophosphamide (CAP). Follow up imaging showed a slight regression in size of the previously noted anterior mediastinal mass which now measured 10.2 × 7.2 cm. The patient received a third cycle of neoadjuvant chemotherapy however, the tumor size remained unchanged.

The patient was then scheduled for a total thymectomy through a mid-sternotomy. Intraoperatively, there was a huge hard thymic mass invading the inner surface of the anterior pericardium and the right phrenic nerve, being very close to the left phrenic nerve. It was also attached to the superior vena cava, innominate vein (InV), ascending aorta (AsA) and part of the right middle and right upper lung lobes.

Total thymectomy with anterior pericardial resection was done; the tumor was dissected from the SVC, the InV and the AsA without complications, the right phrenic nerve was resected en bloc with the tumor and part of the pericardium while the left phrenic nerve was dissected from the mass and preserved. The operation was smooth and uneventful. Postoperatively the chest tube collected 2 l of blood and the patient became hypotensive despite receiving two units of packed red blood cells (PRBCs) and intravenous normal saline. The decision was then made to take the patient back to the operation room and an emergency surgical site exploration via reopening of the midline sternotomy was done within 3 h of the first procedure.

The bleeding source was identified from the left internal mammary vessel and was controlled by clipping, cautery and SURGICEL (Ethicon US). During the intraoperative thirty-minute monitoring for blood pressure improvement, the patient developed ventricular tachycardia, and the defibrillator could not be readily started for technical reasons, so the heart became severely distended and developed asystole. Cardiopulmonary resuscitation (CPR) was initiated manually by cardiac massage with intra-cardiac adrenaline injection. Right ventricular rupture occurred with a 3-cm hole that was followed immediately by an attempt of Proline stich repair that failed, so manual massage continued with a hand closure of the defect until the sinus rhythm reverted. Eventually, repair of the defect was successfully achieved with pericardial patch. Meanwhile, the blood loss was compensated by continuous blood infusion. Within 5 h, the patient received a total of thirteen units of PRBCs, eleven units of fresh frozen plasma (FFP) and one unit of single donor platelet. Blood pressure and vital signs improved afterward and the heart returned to a synchronized rhythm.

**Fig. 3:**
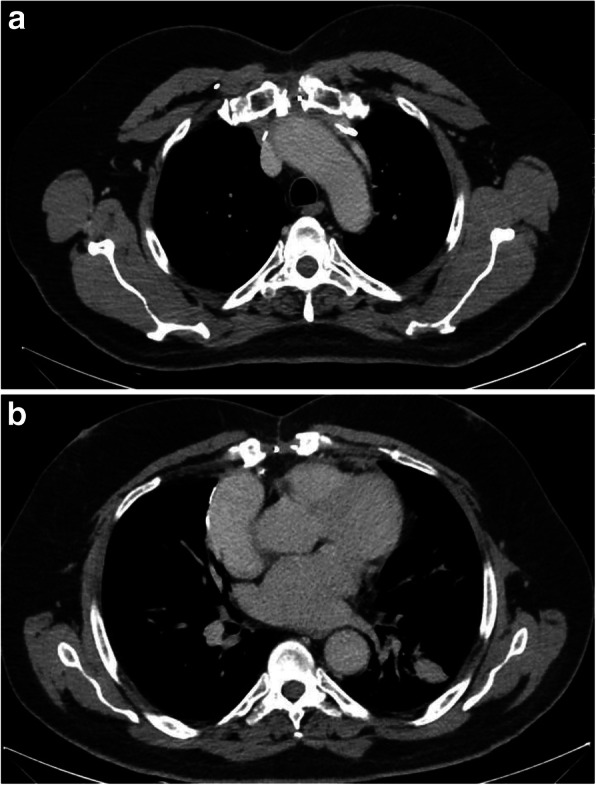
Post-operative chest CT scan, axial view, mediastinal window at the level of the aortic arch (**a**) and the level of the heart (**b**) showing complete excision of the anterior mediastinal mass via midline sternotomy

**Fig. 4:**
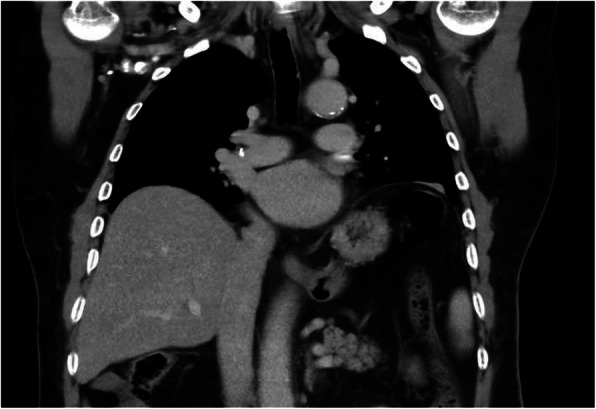
Post-operative chest CT scan, coronal view, mediastinal window showing diaphragmatic elevation

The patient was transferred to the intensive care unit (ICU) on mechanical ventilation for observation. An echocardiogram was done the next day; the left ventricular ejection fraction (EF) was 60% with no evidence of thrombi. On the second post-operative day, the patient developed atrial fibrillation (A.Fib) which was controlled with antiarrhythmic medication (Amiodarone). The patient’s overall status and multi-organ functions returned back to normal during his prolonged ICU stay. However, he remained dependent on ventilator support in spite of multiple trials of weaning. This necessitated a fluoroscopy scan that revealed no appreciable movement in both hemi-diaphragms (Figs. 3, 4), suggesting bilateral phrenic nerve palsy and a decision for elective tracheostomy was made on the 8th post-operative day.

The patient remained completely dependent on the portable mechanical ventilator for a total of 2 months and was discharged from the ICU to the surgical floor 66 days post-operatively with aggressive chest rehabilitation and physiotherapy. He was gradually weaned off ventilation and was discharged on the 85th post-operative day. His tracheostomy tube incision site was closed 5 months after the initial procedure. Two years from the incident, the patient remains in complete remission and is back to his normal daily life with minimal shortness of breath, controlled by an inhaler.

## Discussion

Thymomas are rare tumors with an incidence of 1.5 cases/million individual, with higher incidence reported in African Americans as well as Asians and Pacific Islands which indicates a possible genetic component. It remains however, the most common primary anterior mediastinal tumor [5–8].

They typically occur in adults 40 to 70 years of age, and rarely reported in children and adolescents. Thymoma are generally associated with good outcome and a 5-year survival rate of approximately 90% [8–10]. Usually asymptomatic, some thymoma patients present with chest pain, cough, or dyspnea. Around 30 to 50% of patients with thymomas have myasthenia gravis [9]. Although thymomas can be locally invasive (e.g. pleura, lung), they uncommonly spread to regional lymph nodes or extra thoracic sites [10–13]. Surgery in the form of total thymectomy and complete excision of tumor (R0) is the recommended treatment option [14–16]. Adjuvant radiotherapy is only recommended in cases of incomplete resection [17–20]. or with locally advanced thymomas. Neoadjuvant chemotherapy, using CAP regimen, is advised to be followed by an evaluation for surgery in cases deemed un-resectable initially [18], an effect that was documented in our patient that resulted in the shrinking of the thymic mass.

The first case of cardiac rupture (CR) was described by William Harvey in 1647 [21], and the most common cause of CR is myocardial infarction [22, 23]. Other causes are infective endocarditis, myocardial abscess, dissecting aneurysm of the sinus of Valsalva, syphilitic myocarditis, tuberculosis, echinococcal cyst, trauma and malignancy [24]. Rupture of the free ventricular wall is the most severe, with a 10% incidence in the left ventricle [25]. and much rarer in the right ventricle as reported by Soriano et al. [26], Basarici et al. [27], and De Gennaro et al. [28] as septal rupture associated with right ventricular wall dissection. Niclauss et al. [29] described right ventricular rupture due to mediastinitis.

Cardiorrhexis remains a major cause of death after acute myocardial infarction with a usually delayed diagnosis preventing a chance of successful repair. Once achieved, successful repair of ventricular rupture is followed by long-term survival in the great majority of patients (mean follow-up, 30 months). Thus, attempts to salvage these critically ill patients are worthwhile [30]. Cardiac surgical repair includes two techniques; sutureless, where a patch (Dacron, Teflon, or pericardium) is secured to the infarcted myocardium with the use of tissue adhesive (biological glues or synthetic cyanoacrylate monomers). On the other hand, sutured techniques use a prosthetic patch (Teflon or Dacron) or direct closure under the Cardio-Pulmonary Bypass (CPB) [31]. Several studies published by Padro, Lijoi, and Mariani, who reported 13, 2, and 1 patient respectively, reported ventricular free wall rupture repair due to myocardial infarction without CPB and without any complication post-operatively [32–34].

Our patient suffered from right ventricular wall rupture while attempting to resuscitate him following cardiac asystole. No bypass machine was available at the time of surgery, so manual resuscitation was performed until sinus rhythm reverted followed by primary pericardial patch sutured repair and prolonged ICU (p.ICU) stay that included extensive chest physiotherapy and a strict physiotherapy regimen.

Based on a study on post-operative cardiac patients with p.ICU stay, Rocker et a.l concluded that p.ICU stay beyond 48 h has a potentially increasing risk of long-term mortality [35]. Lei et al. also concluded that extended ICU stay beyond 28 days carries a high mortality rate [36]. Hörer et al. indicated that a higher successful weaning rate was recorded in patients with higher physiotherapy that resulted in greater muscular gain and ended up with early weaning of ventilation [37], an idea that was supported by Nava et al. [38], Carlucci et al. [39], and Martin et al. [40] and that was applied to our patient.

## Conclusion

Thymic mass resection is associated with a risk of bleeding, post-operative wound infection, recurrence, and injury to adjacent structures. We report a case of a 57-year-old previously healthy gentleman, who was diagnosed with an invasive thymoma, treated with chemotherapy followed by surgical resection. Postoperatively he spent more than 2 months in the ICU following an incidence of cardiac rupture during an attempt of CPR. Being a tertiary cancer center, we do not have the equipment needed to perform cardiac bypass surgeries in the event of cardiac rupture. We were forced to use basic techniques in order to save our patient’s life which we enhanced by vigorous and yet adequate chest and whole body physiotherapy that we believe resulted in his fast recovery and returning to his normal life.

## Data Availability

Not applicable.

## Abbreviations

CAP: Cisplatin, Doxorubicin, Cyclophosphamide
CPR: Cardiopulmonary resuscitation
ICU: Intensive care unit
KHCC: King Hussein Cancer Center
CT: Computed tomography
SVC: Superior vena cava
InV: Innominate vein
AsA: Ascending aorta
PRBCs: Packed red blood cells
FFP: Fresh frozen plasma
EF: Ejection fraction
A.Fib: Atrial fibrillation
CR: Cardiac rupture
p.ICU stay: Prolonged ICU stay

## References

[1] Marx A, Chan JK, Coindre JM, Detterbeck F, Girard N, Harris N (2015). The 2015 World Health Organization classification of tumors of the Thymus: continuity and changes. J Thorac Oncol.

[2] Travis WD, Brambilla E, Burke AP, Marx A, & Nicholson AG. (2015). Introduction to the 2015 World Health Organization classification of tumors of the lung, pleura, thymus, and heart. J Thorac Oncol. 2015;10(9):1240-2. 10.1097/JTO.0000000000000663 26291007

[3] National Comprehensive Cancer Network. Nccn.org. 2020. Available from: https://www.nccn.org/professionals/physician_gls/pdf/thymic.pdf [cited 02 Feb 2020].

[4] Yano M, Sasaki H, Moriyama S, Hikosaka Y, Yokota K, Masaoka A (2011). Number of recurrent lesions is a prognostic factor in recurrent thymoma. Interact Cardiovasc Thorac Surg.

[5] Engels E (2010). Epidemiology of Thymoma and associated malignancies. J Thorac Oncol.

[6] Proceedings of the First International Conference on Thymic Malignancies. August 20–21, 2009. Bethesda, Maryland, USA. - PubMed - NCBI [Internet]. Ncbi.nlm.nih.gov. 2020. Available from: https://www.ncbi.nlm.nih.gov/pubmed/21275152. [Cited 02 Feb 2020]. 21275152

[7] Strollo D, de Christenson M, Jett J (1997). Primary mediastinal tumors. Part 1. Chest..

[8] Engels E, Pfeiffer R (2003). Malignant thymoma in the United States: demographic patterns in incidence and associations with subsequent malignancies. Int J Cancer.

[9] Yamada Y, Yoshino I, Nakajima J, Miyoshi S, Ohnuki T, Suzuki M (2015). Surgical outcomes of patients with stage III Thymoma in the Japanese Nationwide database. Ann Thorac Surg.

[10] Masaoka A (2010). Staging system of Thymoma. J Thorac Oncol.

[11] Lewis J, Wick M, Scheithauer B, Bernatz P, Taylor W (1987). Thymoma. A clinicopathologic review. Cancer..

[12] Benveniste M, Korst R, Rajan A, Detterbeck F, Marom E (2014). A practical guide from the International Thymic Malignancy Interest Group (ITMIG) regarding the radiographic assessment of treatment response of Thymic epithelial tumors using modified RECIST criteria. J Thorac Oncol.

[13] Narm KS, Lee CY, Do YW, Jung HS, Byun GE, Lee JG (2016). Limited thymectomy as a potential alternative treatment option for early-stage thymoma: a multi-institutional propensity-matched study. Lung Cancer.

[14] Detterbeck FC, Zeeshan A (2013). Thymoma: current diagnosis and treatment. Chin Med J.

[15] Bretti S, Berruti A, Loddo C, Sperone P, Casadio C, Tessa M (2004). Multimodal management of stages III–IVa malignant thymoma. Lung Cancer.

[16] Potzger T, Sziklavari Z, Diez C, Neu R, Schalke B, Hofmann H (2013). Extended surgical resections of advanced Thymoma Masaoka stages III and IVa facilitate outcome. Thorac Cardiovasc Surg.

[17] Basse C, Thureau S, Bota S, Dansin E, Thomas P, Pichon E (2017). Multidisciplinary tumor board decision making for postoperative radiotherapy in Thymic epithelial tumors: insights from the RYTHMIC prospective cohort. J Thorac Oncol.

[18] Kondo K (2008). Optimal therapy for thymoma. J Med Invest.

[19] Hamaji M, Shah R, Ali S, Bettenhausen A, Lee H, Burt B (2017). A meta-analysis of postoperative radiotherapy for Thymic carcinoma. Ann Thorac Surg.

[20] Forquer J, Rong N, Fakiris A, Loehrer P, Johnstone P (2010). Postoperative radiotherapy after surgical resection of Thymoma: differing roles in localized and regional disease. Int J Radiat Oncol Biol Phys.

[21] Harvey W. Of Circulatio Sanguinis. Exercit 3. Quoted by Morgagni GB In: The seats and causes of diseases. Trad. Benjamin Alexander. London: Letter 27; 1769. p.830.

[22] Freeman WJ (1958). The histologic patterns of ruptured myocardial infarcts. AMA Arch Pathol.

[23] Schechter D (1974). Cardiac structural and functional changes after myocardial infarction. 3. Parietal rupture and pseudoaneurysm. N Y State J Med.

[24] Lynch R, Edwards J (1970). Pathology of coronary atherosclerosis and its complications. The heart.

[25] Kjeld T, Hassager C, Hjortdal V (2009). Rupture of free left ventricle wall, septum and papillary muscle in acute myocardial infarction. Ugeskr Laeger.

[26] Soriano C, Pérez-Boscá J, Canovas S, Ridocci F, Federico P, Echanove I (2005). Septal rupture with right ventricular wall dissection after myocardial infarction. Cardiovasc Ultrasound.

[27] Basarici I, Erbasan O, Kemaloglu D, Arslan G, Bayezid O (2010). Exceptional ventricular septal rupture associated with Intramyocardial dissection throughout the right ventricle. Echocardiography..

[28] De Gennaro L, Brunetti N, Ramunni G, Buquicchio F, Corriero F, De Tommasi E (2010). Septal rupture with right ventricular wall dissecting haematoma communicating with left ventricle after inferior myocardial infarction. Eur J Echocardiograph.

[29] Niclauss L, Delay D, Stumpe F (2010). Right ventricular rupture due to recurrent mediastinal infection with a closed chest. Interact Cardiovasc Thorac Surg.

[30] Bashour T, Kabbani S, Ellertson D, Crew J, Hanna E (1983). Surgical salvage of heart rupture: report of two cases and review of the literature. Ann Thorac Surg.

[31] Iemura J, Oku H, Otaki M, Kitayama H, Inoue T, Kaneda T (2001). Surgical strategy for left ventricular free wall rupture after acute myocardial infarction. Ann Thorac Surg.

[32] Padro JM, Mesa JM, Silvestre J, Larrea JL, Caralps JM, Cerron F, Aris A (1993). Subacute cardiac rupture: repair with a sutureless technique. Ann Thorac Surg.

[33] Lijoi A, Scarano F, Parodi E, Dottori V, Secchi GL, Delfino R, Tallone M, Venere G (1996). Subacute left ventricular free wall rupture complicating acute myocardial infarction. Successful surgical repair with a sutureless technique. Cardiovasc Surg (Torino).

[34] Mariani MA, D'Alfonso A, Nardi C, Grandjean JG (2002). Left ventricular free wall rupture: off-pump sutureless patch and glue technique. Ital Heart J.

[35] Rocker G, Cook D, Sjokvist P, Weaver B, Finfer S, McDonald E (2004). Clinician predictions of intensive care unit mortality*. Crit Care Med.

[36] Lei Q, Chen L, Jin M, Ji H, Yu Q, Cheng W (2009). Preoperative and intraoperative risk factors for prolonged intensive care unit stay after aortic arch surgery. J Cardiothorac Vasc Anesth.

[37] Hörer J, Eicken A, Müller S, Schreiber C, Cleuziou J, Prodan Z (2008). Risk factors for prolonged intensive care treatment following atrial septal defect closure in adults. Int J Cardiol.

[38] Nava S (1998). Rehabilitation of patients admitted to a respiratory intensive care unit. Arch Phys Med Rehabil.

[39] Carlucci A, Ceriana P, Prinianakis G, Fanfulla F, Colombo R, Nava S (2009). Determinants of weaning success in patients with prolonged mechanical ventilation. Crit Care.

[40] Martin A, Smith B, Davenport P, Harman E, Gonzalez-Rothi R, Baz M (2011). Inspiratory muscle strength training improves weaning outcome in failure to wean patients: a randomized trial. Crit Care.

